# Adaptability of the *Saccharomyces cerevisiae* yeasts to wine fermentation conditions relies on their strong ability to consume nitrogen

**DOI:** 10.1371/journal.pone.0192383

**Published:** 2018-02-12

**Authors:** Claire Brice, Francisco A. Cubillos, Sylvie Dequin, Carole Camarasa, Claudio Martínez

**Affiliations:** 1 Centro de Estudios en Ciencia y Tecnología de Alimentos (CECTA), Universidad de Santiago de Chile (USACH), Santiago, Chile; 2 Departamento de Ciencia y Tecnología de los Alimentos, Universidad de Santiago de Chile (USACH), Santiago, Chile; 3 Millennium Institute for Integrative Systems and Synthetic Biology (MII-SSB), Santiago, Chile; 4 UMR SPO: INRA, Université Montpellier, Montpellier SupAgro, Montpellier, France; University of Strasbourg, FRANCE

## Abstract

*Saccharomyces cerevisiae* strains are genetically diverse, largely as a result of human efforts to develop strains specifically adapted to various fermentation processes. These adaptive pressures from various ecological niches have generated behavioral differences among these strains, particularly in terms of their nitrogen consumption capacities. In this work, we characterize this phenotype by the specific quantity of nitrogen consumed under oenological fermentation conditions using a new approach. Indeed, unlike previous studies, our experiments were conducted in an environment containing excess nitrogen, eliminating the nitrogen limitation/starvation factor that is generally observed in fermentation processes. Using these conditions, we evaluated differences in the nitrogen consumption capacities for a set of five strains from diverse origins. The strains presented extremely different phenotypes and variations in their capacities to take up nitrogen from a wine fermentation environment. These variations reflect the differences in the nitrogen uptake capacities between wine and non-wine strains. Finally, the strains differed in their ability to adapt to the nitrogen composition of the environment, leading to variations in the cellular stress states, fermentation performances and the activity of the nitrogen sensing signaling pathway.

## Introduction

The budding yeast *Saccharomyces cerevisiae* is the most exploited microorganism in the food industry because of its ability to achieve complete fermentation of solutions with high sugar contents, and the sugars are converted into alcohol, carbon dioxide and secondary end-products. Different studies have demonstrated the wide genetic diversity of *S*. *cerevisiae* strains [1–4] that result from the combination of their natural genetic diversity (or non-domesticated yeasts) and different domestication processes. Yeasts occupy diverse natural habitats, such as rotten fruits, flowering plant nectars, hops and tree exudates [5–6]. The selective pressure imposed by these stressful environmental conditions has clearly impacted the evolution of these species. Furthermore, human activity has shaped the genetics of the yeast population to obtain yeasts with adaptive properties for use in several industrial fermentation processes, such as baking, brewing, winemaking and the production of various fermented beverages [7–9]. Consequently, *S*. *cerevisiae* strains can be classified in five distinct lineages according to their geographic origin or isolation sources (Saccharomyces Genome Resequencing Project (SGRP)); [10], some of them (wine and sake lineages) are the result of distinct domestication events [11].

Yeasts are continually challenged by stressful environments due to inadequate temperature, lack of oxygen, acidity of the medium or undesirable nutritional composition such as limited amounts of nitrogen, lipids, vitamins or mineral salts. In particular, one of the greatest challenges for yeasts is to cope with low nitrogen availability in their environments, which may result in adaptive evolution [12]. Nitrogen concentrations and compositions vary greatly among musts and various microhabitats. Grape musts and Chinese rice wines both have very high concentrations of amino acids, but the proportions of each amino acid are very different in these two media: glutamine is one of the most abundant nitrogen sources in grape juice and conversely one of the lowest in Chinese rice wine [13–14]. In the same way, the amino acid compositions of floral nectars vary substantially depending on the plant species [15–16].

In the case of winemaking, yeast assimilable nitrogen (YAN) from grape musts is composed of a complex mixture of ammonium ions and amino acids in concentrations between 60 and 2400 mg L^-1^ depending on the grape variety and plant cultivation conditions [17]. Nitrogen is a critical nutrient that directly affects yeast growth [18], fermentation performance [19–21] and the development of organoleptic qualities [22–23]. In particular, it is a key factor for complete the fermentation process [17], and low nitrogen levels in musts may cause slow or stuck fermentations [24]. Yeasts can use thirty distinct nitrogen-containing compounds, which all have different effects on its growth. As a result, nitrogen sources have been classified as preferred (ammonium, glutamine, glutamate and asparagine) or not-preferred (urea, proline and allantoin) [25]. When provided as a mixture, the N-containing compounds are consumed sequentially during the growth phase of wine fermentation. Interestingly, this sequential assimilation slightly depends on the availability of substrates and on the selected strain but is likely the result of differential regulation of the permeases involved in the uptake of these molecules [26]. It is generally accepted that nitrogen sources characterized as preferred present a significate impact on the cellular growth rate during fermentation using a medium with a single nitrogen source. These preferred sources are consumed first, with an exception for ammonium. In the case of ammonium, this source corresponds to a preferred source i.e. one that support optimal growth for which the consumption starts when two other preferred sources as glutamate and glutamine are exhausted [27].

During fermentation, different YAN compounds are transported by various permeases with specific features (such as specificity and affinity). In particular, the three ammonium permeases, Mep1p, Mep2p and Mep3p, have different kinetics properties depending on the ammonium concentration (K_m_ values for NH_4_^+^ of 10 μM, 1 μM and 2 mM for *MEP1*, *MEP2* and *MEP3*, respectively) [27–28]. Amino acids enter the cells via either permeases specific to one or more amino acids [26] or via a general amino acid permease (such as Gap1p and Agp1p), which can transport amino acids unselectively [29]. The activities of these transporters are strongly regulated by a complex regulatory network and depend on the quantity and quality of the nitrogen sources in the must [30–31]. First, the Ssy1-Ptr3-Ssy5 (SPS) amino acid sensing pathway [32] is expressed at the start of nitrogen consumption [33]. It can detect the presence of specific amino acids in musts, including aspartic and glutamic acid, threonine, glutamine, leucine, methionine, isoleucine, serine, phenylalanine, valine, tryptophan and tyrosine, which will induce the transcription of genes encoding a subset of AAP (amino acid permease) genes [34]. At the same time, the nitrogen catabolite repression (NCR) regulatory system, which involves two positive regulatory factors, Gln3p and Gat1p [35, 30**]**, prevents the transcription of genes coding for transporters of nitrogen sources consumed in the late stage of growth, including the *MEP* genes. In addition, at the end of the growth phase when nitrogen becomes limiting, the general amino acids control (GAAC) process is activated, slowing the transcription in progress and inducing the transcription of new genes in response to the limited nitrogen [36]. Both the NCR and GAAC processes are controlled by the TOR pathway [37].

Within *S*. *cerevisiae* species, important variations have been reported in the ability of the strains to efficiently uptake YAN [26], in their nitrogen consumption profiles [38–39, 12**]** and in their preferences for nitrogen sources [40–42, 12]. These diverse phenotypes are likely the result of variations in the regulation mechanisms of nitrogen uptake between strains [41, 43]. In line with this hypothesis, several allelic variants involved in the differences in the nitrogen consumption among strains have been recently identified; these include genes related to amino acid transporters (*VBA3*, *GLT1*, and *AGP1*) and genes involved in nitrogen sensing and signaling (*ASI1*, *ASI2*, *PDC1*, *GCN1*, and *MDS3*) [39,43–45]. However, the underlying mechanism of the difference in nitrogen consumption between strains remains unknown.

The aim of this study was to determine the molecular mechanisms associated with the variations in nitrogen consumption capacity between *S*. *cerevisiae* strains. We first explored the profiles of the consumption of nitrogen sources of strains from different habitats (wine, sake, rum, fruit, and soil) during wine fermentations carried out in a synthetic grape juice containing an excess of nitrogen to avoid the molecular responses related to nitrogen starvation. This comparison revealed differences between the abilities of the strains to efficiently uptake some N-containing compounds, and the molecular basis for the discrepancies was further investigated using reciprocal hemizygosity and transcriptomic analysis.

## Materials and methods

### Yeasts strains and culture conditions

The five *S*. *cerevisiae* strains used in this study are listed in Table 1. All the strains used here are haploid with different geographical origins, and three (WE, FWI and SA) are used in different industrial processes. The haploid parental strains used in this study, namely, North American (**NA**; YPS128, *MAT a*, *ho*:*HygMX*, *ura3*::*kanMX*), West African (**WA**; DBVPG6044, *MAT a*, *ho*:*HygMX*, *ura3*::*kanMX*) and Sake (**SA**; Y12, *MAT alpha*, *ho*:*HygMX*, *ura3*::*kanMX*), have been previously described [46–47]. The haploid Wine/European strain (**WE**; 59A, *MAT a*, *ho*) and the Rum strain (**FWI**; A390D2, French West Indies) were previously described by Ambrosset et al. [48] and Marsit et al. [49], respectively. The strains were stored and plated on yeast extract-peptone-dextrose (YEPD) medium for single-colony isolation.

**Table 1:** *Saccharomyces cerevisiae* strains used in this study.

Identification	Lineages	Geographic origins	Utilization-Isolation	References
WE (59A)	Wine/European	Europe	Oenological wine fermentation	*Ambrosset* et al. *2011*
FWI (A390D2)	Mosaic	French West Indies	Sugar cane juice and molasses	*Marsit* et al. *2015*
SA (Y12)	Sake	Africa	Rice wine fermentation	*Liti* et al. *2009*
WA (DBVPG6044)	West African	Africa	Fermenting fruit juices	*Liti* et al. *2009*
NA (YPS128)	North American	Americas	Soil beneath OAK	*Liti* et al. *2009*

### Fermentation conditions

All cultures were developed from a pre-culture started from a single colony grown overnight in synthetic medium (SM) at 25°C with constant shaking (280 rpm). One aliquot from this pre-culture was used for inoculation at a density of 1x10^6^ cells mL^-1^. The yeast strain was cultured in the SM as described by Bely et al. [50]; the medium contained 200 g L^-1^ glucose with various nitrogen concentrations. Briefly, SM465 was supplemented with a final concentration of 465 mg L^-1^ of assimilable nitrogen (YAN) corresponding to 161 mg L^-1^ ammonium ion and 302 mg L^-1^ of a mixture of 19 amino acids (636.1 mg L^-1^ L-proline, 505.3 mg L^-1^ L-glutamine, 374.3 mg L^-1^ L-arginine monohydrochloride, 179.3 mg L^-1^ L-tryptophan, 145.3 mg L^-1^ L-alanine, 120.4 mg L^-1^ L-glutamic acid, 78.5 mg L^-1^ L-serine, 75.92 mg L^-1^ L-threonine, 48.4 mg L^-1^ L-leucine, 44.5 mg L^-1^ L-aspartic acid, 44.5 mg L^-1^ L-valine, 37.9 mg L^-1^ L-phenylalanine, 32.7 mg L^-1^ L-isoleucine, 32.7 mg L^-1^ L-histidine monohydrochloride monohydrate, 31.4 mg L^-1^ L-methionine, 18.3 mg L^-1^ L-tyrosine, 18.3 mg L^-1^ L-glycine, 17.0 mg L^-1^ L-lysine monohydrochloride, and 13.1 mg L^-1^ L-cysteine).

The phenotypic parameters were measured in microfermenters (with 80 mL of SM) with an airlock system, and the mixtures were stirred each day. Microfermentations were carried out in isothermal conditions (25°C) with manual measurements to determine the CO_2_ released. The amounts of CO_2_ lost (g/L) from the fermentations are available in S1 Table. To obtain full fermentation kinetics, 1.2-liter fermenters (containing 1 liter of medium) with airlocks to maintain anaerobiosis were used. The amount of CO_2_ released during fermentation was calculated from automatic measurements (taken every 20 min) of the fermenter mass [51]. The CO_2_ production rate was estimated using a sliding-window second-order polynomial fitting of the last ten measurements of fermenter weight. The use of this technique for monitoring fermentations has already been validated and is thoroughly described by Bezenger et al. [52–53]. For each strain, three fermentations were conducted in parallel in three distinct fermenters. Each strain was pre-cultured in triplicate (one by fermentation), and the same lot of synthetic medium was used for all pre-cultures and fermentations.

### Analytical methods

The yeast population was estimated after cell sonication using an electronic particle counter (Multisizer 3 Coulter Counter; Beckman Coulter) and by dry weight determination. The residual amino acid and ammonium concentrations were determined by HPLC. Samples were collected at 24-hour intervals after inoculation for the first 72 hours and then every 48 hours until the fermentation was completed. HPLC data are presented in S2 Table. For each sample, 1 mL of MS was centrifuged at 12,000 rpm for 10 min, and the supernatant was collected. A 20-μl aliquot of supernatant was injected into a Prominence HPLC system (Shimadzu, USA) equipped with a Biorad HPX-87H column according to Nissen et al. [54]. The concentrations of ammonium ion and the 14 amino acids were measured using the previously described HPLC analysis method [55]. The consumption of each nitrogen source was estimated from the difference between the initial and final amounts of each source before and after fermentation. In this study, YAN estimation did not consider four amino acids (His, Gly, Cis and Lys) because the HPLC measurements of these compounds are not sufficiently accurate, and they represent a small percentage of the total YAN. In addition, proline is poorly consumed during wine fermentation that occurs under anaerobiosis since its metabolism involves an O_2_-dependent oxidase [56]. Consequently, this amino acid was not considered in the study.

Total cell protein was determined by the bicinchoninic acid protein assay (BCA), which is a modified biuret method. Briefly, proteins were extracted from cells by incubation with 50% (vol/vol) dimethylsulfoxide for 1 hour at 100°C and then quantified with the BCA kit (Sigma-Aldrich, France). Bovine serum albumin (BSA) was used as the calibration standard.

The different values obtained were compared using Fisher’s LSD test and each mean value and standard error of the mean (SEM) were calculated from three replicates.

The maximum uptake rate (μMax), i.e. the maximum flow of nitrogen consumed observed during fermentation process (mg N L^-1^ h^-1^)was determined using GraphPad Prism version 6.0 for Windows, GraphPad Software, La Jolla California USA, www.graphpad.com, according to the following equation:

$$\mu Max=\frac{Vmax\times X^{h}}{Khalf^{h}\times X^{h}}$$

where the X parameter corresponds to the time value presented in abscissa, and the values of Vmax, h and Khal are provided by the software.

### Functional validation

Permease sequences were compared using SIFT analysis [57]. Two strains were used to perform the reciprocal hemizygosity assay on the *TAT2* and *DIP5* genes, WE and SA strains, which are representative of the extreme phenotypes (strong and weak nitrogen consumption, respectively). Each construction was obtained by crossing a strain bearing an inactivated form of the gene with one containing a functional form. Yeasts were transformed as described by Guldener et al. [58], and hemizygotes were tested on YPD plates supplemented with G418 and hygromycin (100 mg/mL G418 and 100 mg/mL hygromycin). The deletions of the target genes were confirmed by PCR using primers positioned upstream or downstream of the coding region with a primer in the cassette insertion.

Sequence comparison demonstrated that the *RPI1* gene causes sequence truncation in wine-type strains [46], like the WE strain used in this study. For the assay performed on the *RPI1* gene, yeasts were crossed to generate the reciprocal hemizygote strains and selected on double drug plates (50 mg/mL hygromycin and 100 mg/ml nourseothricin). The deletions of the target genes were confirmed by PCR using the primer pairs A1/S8 or A4/S5 [59]. The diploid hybrid strains were confirmed by *MAT* locus PCR [60].

Comparison of the residual amino acids and ammonium consumption for the reciprocal hemizygotes was determined at the end of fermentation by HPLC (see S3 Table).

### RNA extraction, library preparation, Illumina sequencing and RNA-seq analysis

Two strains with extreme phenotypes were subjected to gene expression analysis, i.e., Wine/European and Sake representing high and low nitrogen consumption capacities, respectively. Three biological replicates were fermented in SM465 as previously described, and cell cultures was collected at two conditions; total RNA was obtained from each culture when 15% of the YAN had been consumed by the two strains (corresponding to 24 hours) and when the two strains reached their maximum YAN consumed (at 45 g of CO_2_ released) utilizing the TRIzol method described by Chomczynski and Sacchi [61]. For each RNA extraction, 1×10^9^ cells were pelleted by centrifugation (3000 rpm, 2 min) and mechanically lysed by vortexing for 8 min with glass beads (diameter = 0.3 mm) in 400 μl of TRIzol (Gibco BRL) at 4°C. The liquid phase was collected, and TRIzol was used to dilute the sample to a final volume of 4 mL. Samples were incubated for 5 min at room temperature and 800 μl of chloroform was added. The mixture was vortexed, incubated for 2 min at room temperature, and then centrifuged (10,000 rpm, 15 min). An equal volume of cold (−20°C) isopropanol was added to the aqueous phase to precipitate the RNAs. The samples were then incubated for 10 min at room temperature and centrifuged (10,000 rpm, 10 min). The resulting nucleic acid pellet was washed twice with 2 mL of 75% ethanol–diethyl pyrocarbonate-treated water and then dissolved in 150 μl of nuclease-free water (Qiagen). The RNAs were stored in 100 μg aliquots of these preparations and were purified with an RNeasy mini kit (Qiagen) following the RNA cleanup protocol including membrane DNase digestion.

RNA-seq was performed as previously described [45]. Briefly, mRNA from 1 μg of the total RNA was enriched using magnetic beads for mRNA purification. Enriched mRNAs were eluted and fragmented at 94°C for 5 min. The double-stranded cDNA was acquired by RT-PCR using the above fragmented mRNA followed by end-repair, single A base adding and adapter/index ligation. The ligation product was amplified by PCR. The size of the end product was approximately 260 bp, and sequencing was conducted on an Illumina HiSeq^™^4000 (Illumina Inc, San Diego, CA, USA) in paired-end mode with a read length of 100 bp. The quality of the raw reads was assessed using the FASTQC tool kit (http://www.bioinformatics.babraham.ac.uk/projects/fastqc/). Low quality reads were discarded after Trimmomatic screening with default score settings and a phred score cut-off of 30 (http://www.usadellab.org/cms/?page=trimmomatic). RNA-seq reads were then aligned to the S288c reference (*S*. *cerevisiae* genome obtained on 03/03/2016, from the Saccharomyces Genome Database, FTP SITE: http://downloads.yeastgenome.org/sequence/S288C_reference/genome_releases/ corresponding to a stable release from January 2015) using TopHat with default score settings [62]. BAM files were sorted and indexed using Samtools with default score settings [63]. Alignments were then processed, and gene counts were obtained using HTSeq [64] with the no-stranded and -gene counts configuration from the S288c gff file. Differential expression was assessed with edgeR utilizing the exactTest function [65]. The results obtained from edgeR provided the differentially expressed genes between strains and/or conditions with FDR < 0.05. FDR was estimated utilizing the default Benjamin-Hochberg correction [66].

The gene ontology (GO) technique was used to group genes with differing expressions using the Funspec program with the Bonferroni correction at a P value cutoff of 0.05 [67].

### Data access

The complete data sets of the RNA-Seq are deposited at the National Center for Biotechnology Information (NCBI) BioProject (http://www.ncbi.nlm.nih.gov/bioproject/), under BioProject PRJNA39755.

## Results

### Nitrogen consumption profiles of the five strains during fermentation

**Fig 1:**
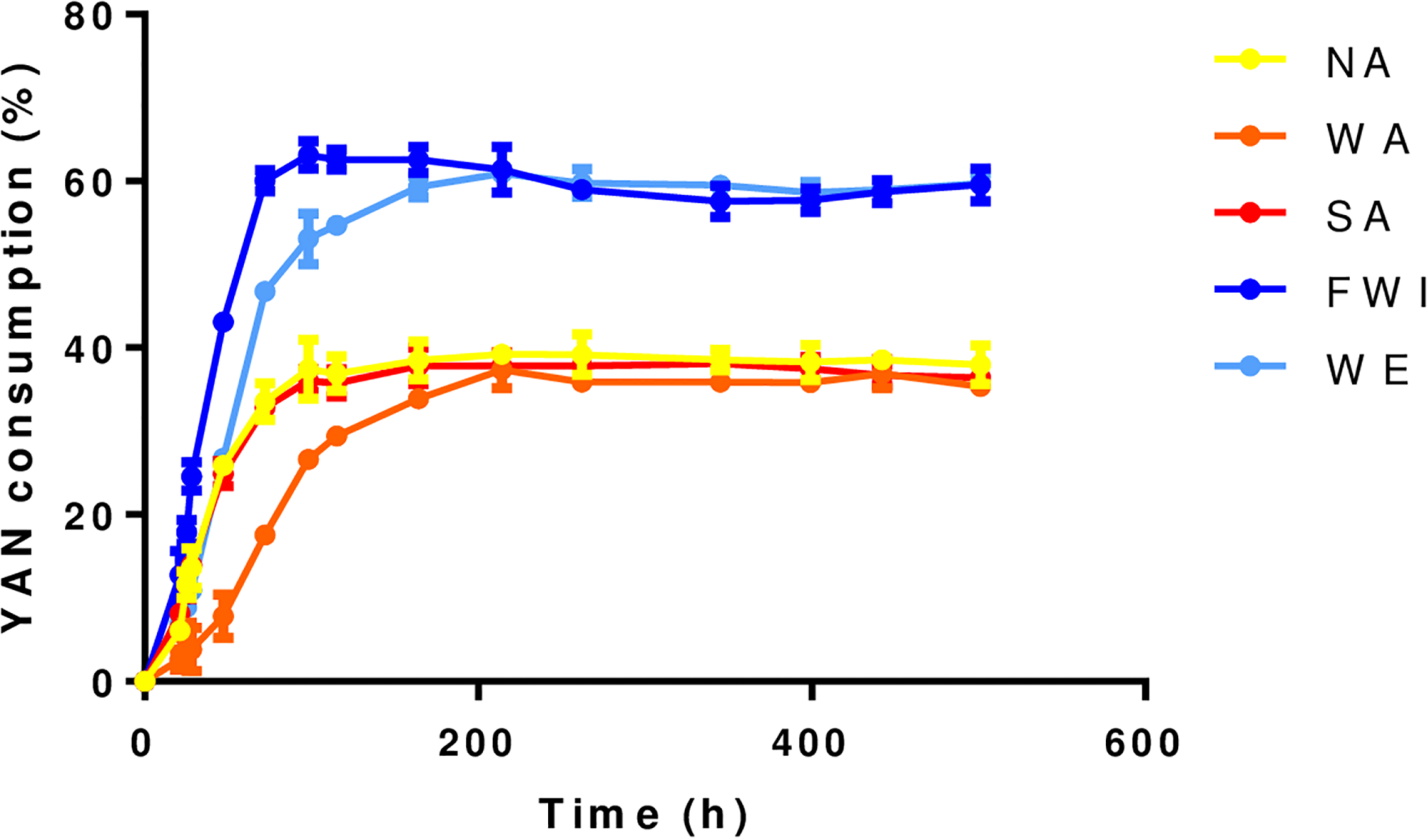
Comparison of the kinetic profiles of YAN consumption by the five strains. The YAN consumption is expressed as a percentage based on the amount of nitrogen concentration consumed by the yeast by the end of the fermentation. Mean values and mean standard errors were calculated from three replicates.

**Fig 2:**
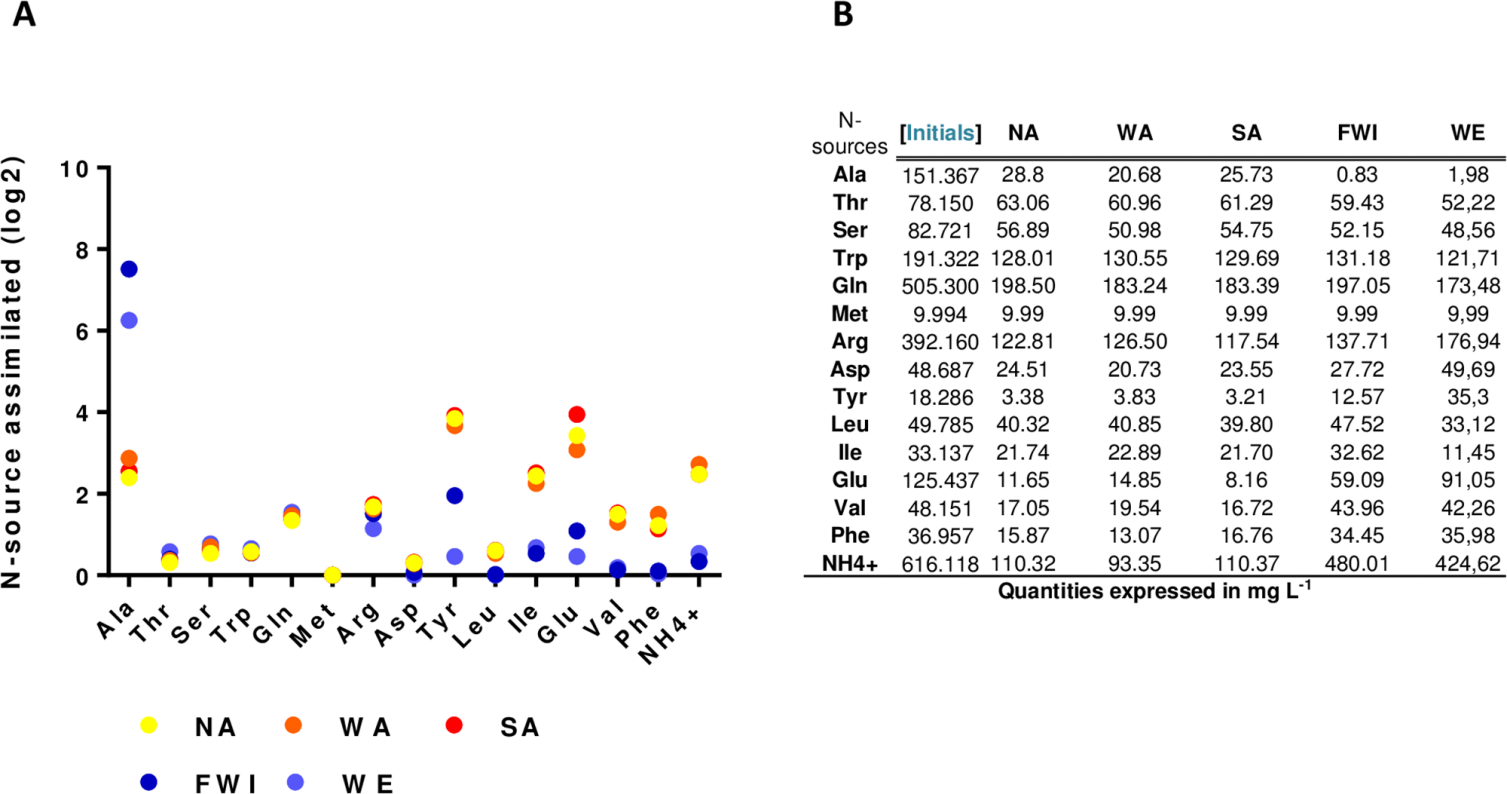
Profiles of the residual N sources at the end of fermentation. (A) Comparison between strains in terms of the 14 residual amino acids and ammonium they did not consume. Values are expressed in log2 of the N sources not consumed in comparison to the amount of nitrogen initially in the MS465. (B) The quantity of each residual nitrogen source expressed in mg L^-1^.

To investigate the variations in YAN consumption capacity between strains, we compared five *S*. *cerevisiae* isolates from distinct ecological backgrounds (Table 1); the NA and WA strains originated from natural habitats (fruit and soil, respectively), while WE, FWI and SA were isolated from industrial environments (wine, sugar cane and rice fermentation, respectively). The abilities of these strains to consume the 14 amino acids and ammonium sources were first evaluated under wine fermentation conditions. The stressful environment used in winemaking, which is characterized by high sugar and ethanol concentrations, low pH and anaerobiosis, constitutes a model system for studying yeasts with diverse phenotypic characteristics [68]. All fermentations were carried out in triplicate in a synthetic medium (SM465) containing 240 g L^-1^ sugars and 465 mg N L^-1^. Under these conditions, nitrogen was provided in excess to override the nitrogen limitation responses, and the yeast growth stopped as a result of limitations in other nutrients (lipids or vitamins) [69–70]. The comparison of the kinetics of nitrogen consumption revealed the distinctions between the phenotypes (Fig 1) and reflected the differences between the abilities of the strains to efficiently uptake nitrogen. Overall, the WE and FWI strains consumed higher amounts of total YAN (between 1324 and 1334 mg L^-1^) during fermentation than that of the NA, WA and SA (total consumption lower than 853 mg L^-1^). Furthermore, the profiles of the residual nitrogen sources differed substantially between the strains (Fig 2), allowing the nitrogen sources to be classified into three groups. Alanine was the sole amino acid that is more consumed by the NA, WA and SA yeasts. The second group, including threonine, serine, tryptophan, glutamic acid, methionine, arginine and aspartic acid, were the nitrogen compounds consumed to the same extend by the five strains, while tyrosine, leucine, isoleucine, glutamate, valine, phenylalanine and ammonium were more efficiently consumed by the WE and FWI strains.

**Fig 3:**
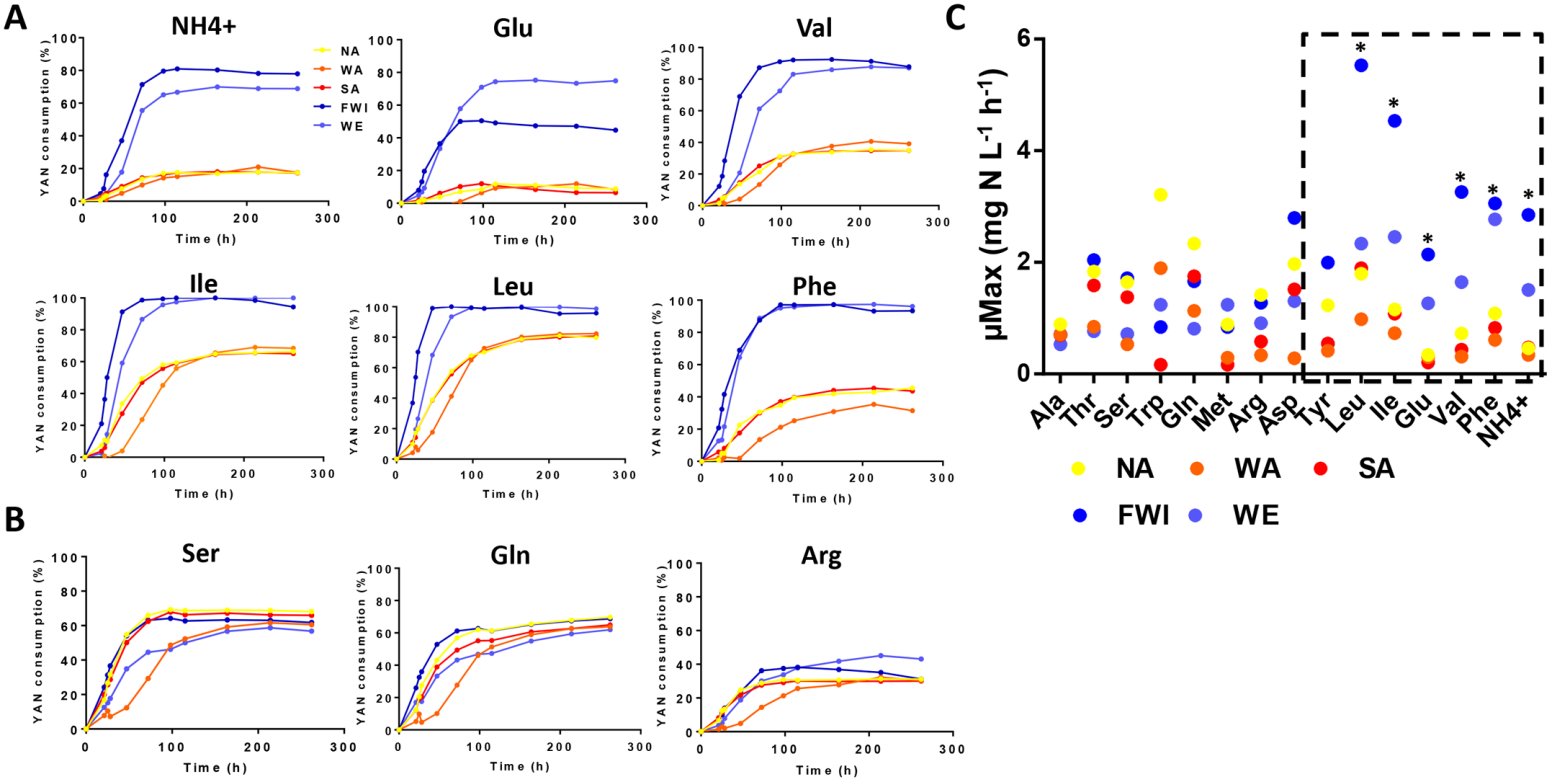
Comparison of the N source consumption dynamics between the strains. (A) Comparison of the consumption kinetics of the N sources differentially consumed between the strains. (B) Comparison of the N sources consumed at equal rates by all five strains. The YAN consumption is expressed as a percentage based on the amount of nitrogen consumed by yeast at the end of the fermentation. (C) Comparison of the maximum uptake rates (μMax values) (mg N L^-1^.h^-1^) of the N sources between the strains. Data within the dashed box correspond to the N sources with the best consumption profiles for the WE and FWI strains. Mean values were calculated from three replicates from each strain. Values marked with an asterisk are significantly different (p*Ile*<0.050; p*Glu*<0.023; p*Val*<0.050; p*Phe*<0.002; and p*NH*_*4*_^+^<0.040).

Weak differences between strains were observed in the profiles and the maximum rates of consumption of the amino acids showing similar residual concentrations at the end of the fermentations (Fig 3). By contrast, the WE and FWI strains displayed significant differences with higher rates of consumption of ammonium, phenylalanine, tyrosine, valine, glutamate, isoleucine and leucine. In particular, the maximum rate of phenylalanine consumption by the WE and FWI strains was close to 2.90 mg N L^-1^.h^-1^, while it was 0.8 mg N L^-1^.h^-1^ for other strains (Fig 3); the WE and FWI consumed ammonium 3.5 and 6.8 times faster, respectively, than the NA, WA and SA strains. Interestingly, some of these nitrogen sources share common transporters, for example, *BAP2*,*3* for isoleucine, valine and leucine and *TAT1*,*2* for tyrosine and phenylalanine [71–72].

Based on these data, we can suggest that differences between the abilities of the strains to uptake specific nitrogen sources, including tyrosine, ammonium, phenylalanine, valine, glutamate, isoleucine and leucine may explain the variations in the amounts and the profiles of the residual YAN at the end of the fermentations carried out under excess nitrogen.

### Functional analysis

To further investigate the differences in the abilities of the WE and FWI on one hand and the SA, WA and NA on the other hand to uptake some specific nitrogen sources, we compared the sequence of the permeases involved in the uptake of these compounds. Indeed, the variations in the transport efficiency could be explained by mutations in the active sites of these proteins. Sequence comparisons revealed that only Tat2p and Dip5 exhibit non-synonymous mutations in their coding sequences (S1A Fig). We carried out a reciprocal hemizygosity analysis for these two genes using the WE and SA strains, which displayed strong and weak abilities, respectively, to transport glutamate, tyrosine and phenylalanine. However, no differences were found in the amount of YAN consumed between the reciprocal hemizygous strains, ruling out the contribution of mutations in the coding sequence to the differential capacity of the strains to uptake some nitrogen sources (S1B Fig).

### Transcriptomic comparison between two strains with different nitrogen consumption capacities

To assess the potential contribution of transcriptomic regulation to the differences in the YAN consumption between the yeast strains, we then examined the gene expression profiles of two strains exhibiting strong and weak abilities (WE and SA, respectively) to uptake some nitrogen compounds. The transcriptomes of the two strains were compared at two specific moments of SM465 fermentation; during the growth phase, when the rate of YAN consumption was at its maximum (when 15% of total YAN was consumed), and at the end of the growth phase, when the nitrogen consumption had stopped.

**Fig 4:**
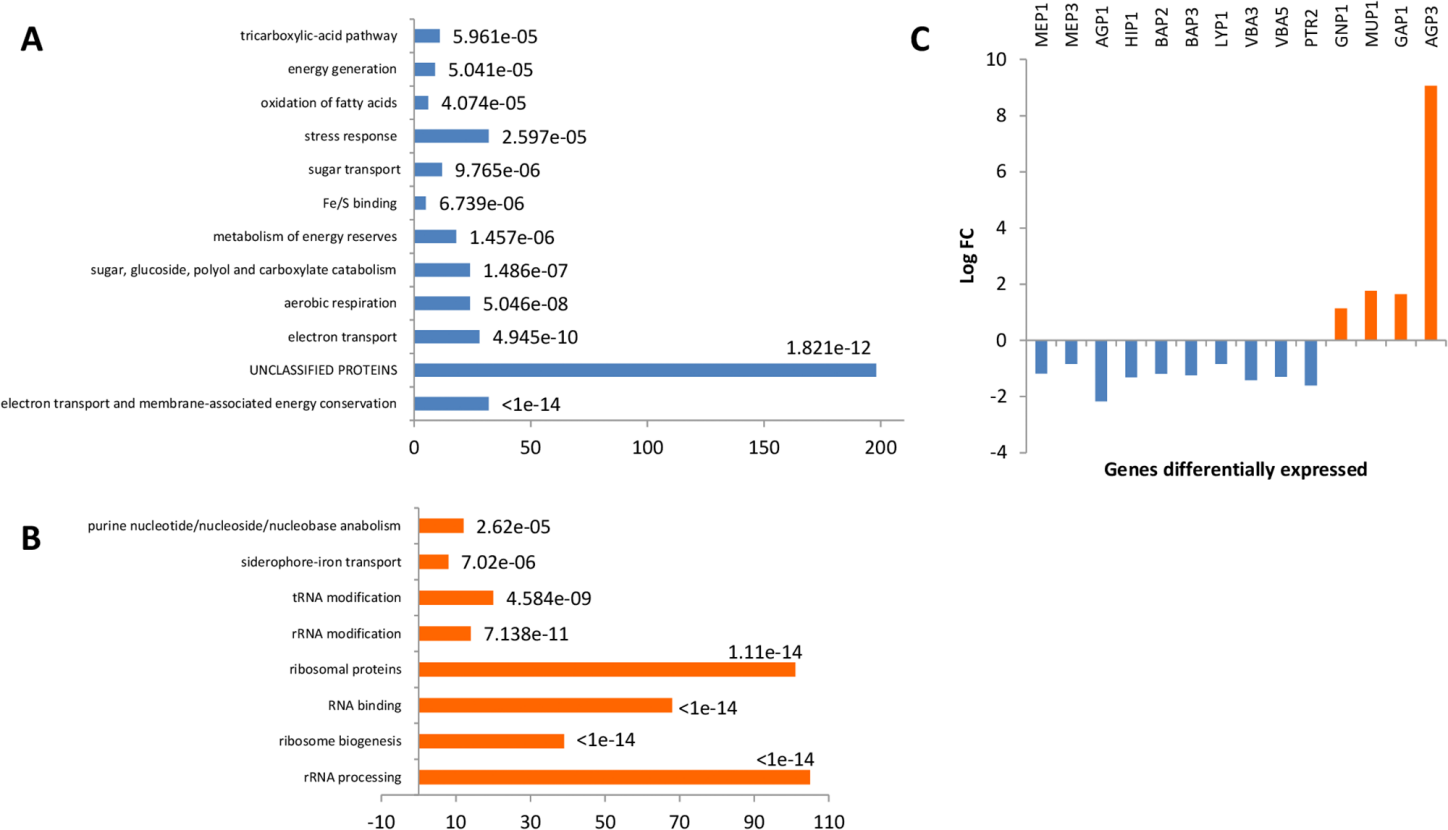
Analysis of transcriptomic data at 15% of YAN consumed (mean of three repetitions). Enrichment functions for the Sake (A) and WE strains (B). The values correspond to the number of genes in the Funspec functional category (Bonferroni correction and P value of 0.05). P values are indicated for each category. (C) Log FC representation of genes involved in nitrogen transport for the WE strain (blue) and SA strain (orange).

During the growth phase, 1,341 genes were differentially expressed with log-fold-change- higher than 1 in the WE and SA strains (Fig 4). The set of 612 genes overexpressed in SA (Fig 4A) was enriched in genes related to stress responses, such as genes encoding for proteins involved in the production of reserve carbohydrates (*TPS2*, *PGM2*, *TLS1*, *GIP2*, *GSY1*, *GYS2*, *GLC3*, *YPI1*, and *GAC1*), genes encoding for stress response proteins that can be expressed in environmental stress conditions (*ALD3*, *DDR2*, *XBP1*, *HSP12*, and *HSP104*) or genes associated with autophagy (*ATG1*, *ATG8*, *ATG15*, *ATG19*, *ATG22*, *ATG32*, and *ATG33*). In addition, we observed an enrichment in genes involved in the tricarboxylic acid cycle (TCA) and genes involved in aerobic respiration, suggesting this strain is under higher stress during the fermentation process [73]. In contrast, the set of 729 genes overexpressed in WE included many genes involved in protein synthesis, ribosome biogenesis and RNA processing and metabolism (Fig 4B). These distinct expression patterns likely reflect the different adaptations of the WE and SA strains to the wine fermentation conditions. Unaccustomed to this stressful environment, the SA strain exhibited an important stress response, while the WE strain, which is more accustomed to these conditions, displayed a higher growth capacity based on the overexpression of genes involved in cell proliferation.

**Fig 5:**
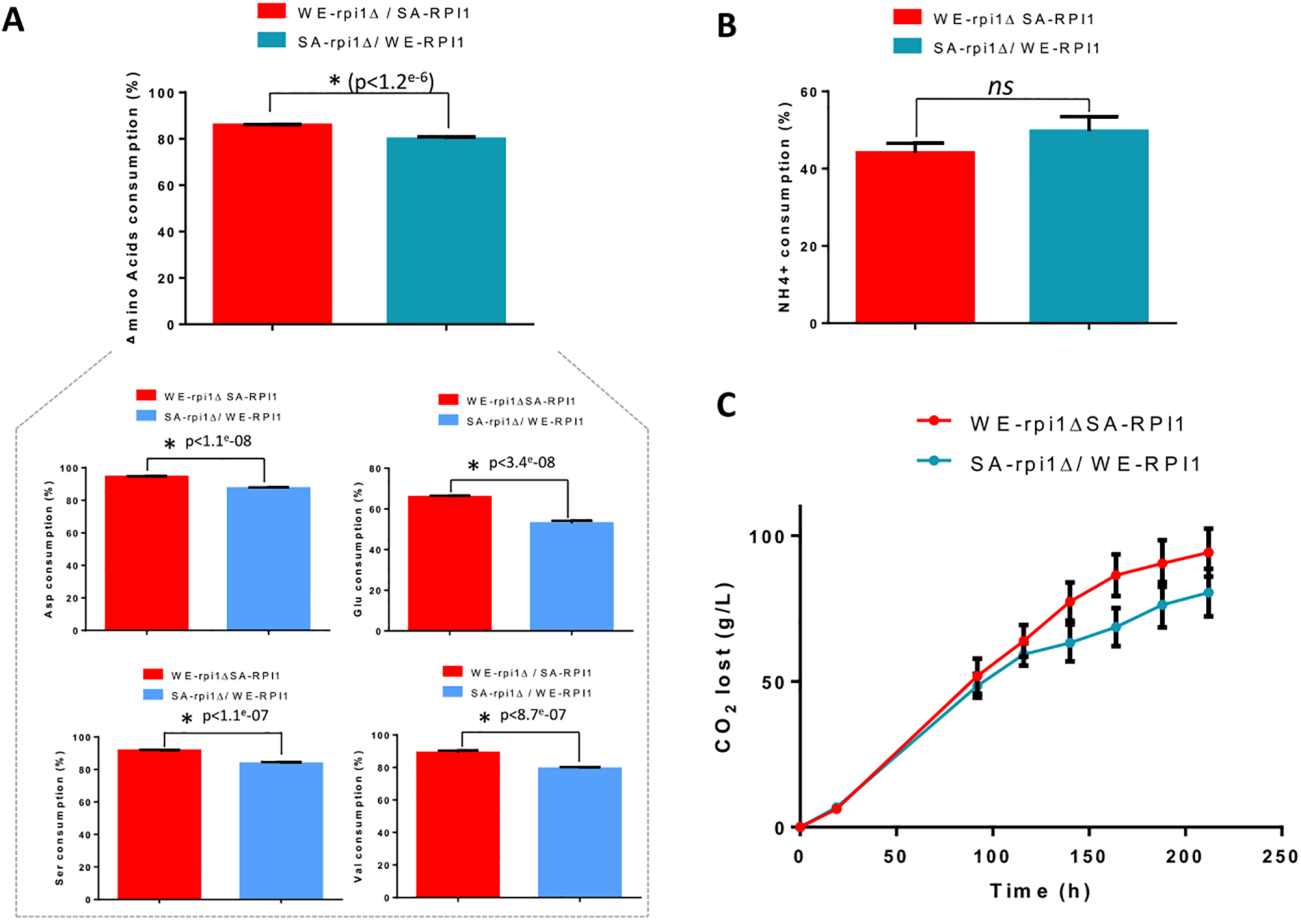
Reciprocal hemizygosity analysis on the RPI1 gene. The levels of amino acids (A) and ammonium (B) consumed by the constructed hemizygotes are expressed as a percentage based on the amount of nitrogen consumed. (C) CO_2_ lost (g/L) comparison. Mean values and mean standard errors were calculated from five replicates. The SEM is indicated by vertical error bars. Values marked with an asterisk are significantly different (p<0.05).

In agreement with the results obtained in a previous study [45], a differential expression of the *RPI1* gene, a transcription factor involved in stress tolerance during fermentation [74], was observed. The characterization of reciprocal hemizygotes obtained by crossing a WE strain with a truncated sequence with an SA strain revealed the impact of *RPI1* on the nitrogen consumption capacity and the fermentation performances (Fig 5). Surprisingly, the SArpi1Δ/WE-RPI1 strain, expressing the allele of the strain with the higher amino acid uptake capacity, showed lower consumptions of aspartic acid, glutamic acid, serine and valine. This effect is opposite of what is seen in the WE strain phenotype and would be consistent with the notion that cellular stress has a greater impact on the SA strain and results in the phenotype having weak nitrogen consumption. Functional analysis also revealed that a decrease in the YAN consumption capacity for the wine strain had repercussions on its fermentation performance, which is in agreement with the phenotype of the five strains.

Focusing on genes involved in nitrogen transport, we found that the WE strain overexpressed most of the genes encoding for cytoplasmic membrane permeases (*MEP1*, *MEP3*, *AGP1*, *HIP1*, *BAP2*, *BAP3*, and *LYP1*), which includes genes involved in the transport of ammonium and branched amino acids as well as the genes encoding for vacuolar basic amino acid transporters (*VBA3*, *VBA5*, and *PTR2*) (Fig 4C). By contrast, the SA strain overexpressed only four genes encoding for permeases, namely, *GNP1*, *MUP1*, *AGP3* and *GAP1*.

**Fig 6:**
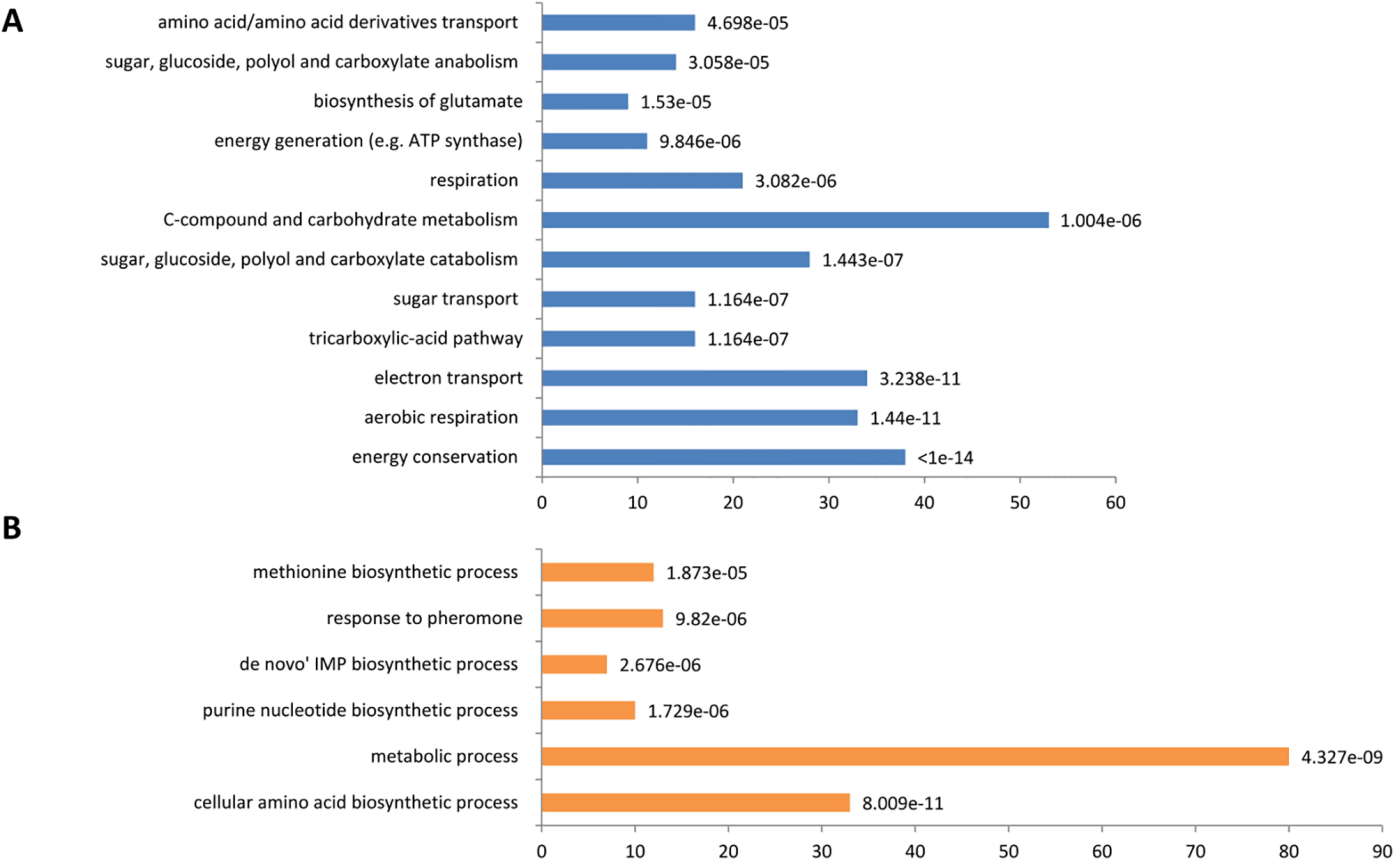
Analysis of the transcriptomic data from the end of the growth phase (mean of three repetitions). Enrichment functions for the SA (A) and WE strains (B). The values correspond to the number of genes in the Funspec functional category (Bonferroni correction and P value of 0.05). P values are indicated for each category.

At the end of the growth phase, 1,463 genes were differentially expressed with log-fold-changes greater than 1 between the SA and WE strains, most of the genes were involved in metabolism; 811 genes were overexpressed in the SA strain and 652 genes in the WE strain. Interestingly, the transcriptomic expression profiles between the two conditions were substantially different, with only 25 and 50% of common genes observed for the WE and SA strains, respectively. The SA strain was enriched in genes involved in sugar transport (*HXT* genes) and, surprisingly, for genes responsible for the uptake of amino acids regulated by either the NCR system (*CAN1*, *ALP1*, *GAP1*, and *PUT4*) or the SPS system (*AGP1*, *AGP2*, *AGP3*, *GNP1*, and *MUP1*) (Fig 6A). Under the same physiological conditions and in contrast with what was seen during the growth phase, the WE strain did not overexpressed genes encoding for permeases, but instead, it overexpressed genes related to cellular amino acid biosynthetic processes (*LYS*, *HIS*, *ARG*, *SER* and *MET* families) and genes involved in de novo IMP biosynthetic processes with the overexpression of the *ADE* family of genes (Fig 6B). The comparison of the transcriptomic patterns revealed distinct physiological states between the SA and WE strains at the end of the growth phase. The WE strain, which efficiently consumed and stored nitrogen during the growth phase [18], redistributed its nitrogen pool towards the anabolism of amino acids required for proteins synthesis. In contrast, relative to the WE strain, the SA strain overexpressed nitrogen permeases to increase its intracellular nitrogen pool.

### Comparison of physiological parameters

Nitrogen consumption is a key factor in the fermentative abilities of yeasts. To investigate the impact of nitrogen consumption on the fermentation performances of the strains, we studied their fermentation profiles in SM465 under oenological conditions (S2 Fig).

Results show that the two classes of strains could be differentiated according to their fermentation kinetics; the WE and FWI strains, which efficiently consume nitrogen sources, presented higher fermentation rates than the NA, WA and SA strains and pronounced reductions in the fermentation duration. It is interesting to note that the WE and FWI strains show similar behaviors in the winemaking process. This can be explained by the fact that these two strains are used in fermentations with high nitrogen concentrations [75] (CTCS Martinique personal communication). Furthermore, the rum fermentation process is technically closer to the winemaking process than the sake making process is to the winemaking process.

Moreover, the WE and FWI strains presented higher biomass productions at the end of fermentation than the other strains (S2 Fig). These results are consistent with the findings of Camarasa et al. [68], suggesting that yeast strains that produce more biomass present have better fermentative abilities.

Nitrogen represents a non-negligible part of the cellular weight [76]. We evaluated whether variations in nitrogen consumption could be associated with variations in the nitrogen content of the yeast cells. We quantified the total protein content at the end of the fermentation process for each studied strain (S2 Fig). Our results indicate that the percentage of protein per biomass is equivalent among the strains. However, the amount of nitrogen used for protein synthesis is higher in the WE and FWI fermentations since the production of biomass is more important under these conditions.

## Discussion

Phenotypic diversity among yeast strains has long been characterized and exploited, particularly with regards to the central carbon metabolism [77]. However, some aspects of the differences in the abilities of strains to efficiently metabolize nitrogen compounds remain unclear. Previous studies have demonstrated the substantial diversity of nitrogen consumption capacities within *S*. *cerevisiae* species [26, 38, 43, 45], which is partly related to their original environment. It has also been reported that the differences between strains can be amplified by providing excess nitrogen [78]. In this study, the comparison of the nitrogen consumption profiles of five *S*. *cerevisiae* strains, originating from nitrogen-poor (NA, WA and SA) or nitrogen-rich (WE, FWI) environments, confirmed these differential behaviors. The WE and FWI strains consumed up to 20% more YAN during fermentations with excess nitrogen than the NA, WA and SA strains. Furthermore, we demonstrated that the differences between the capacities of the strains to consume YAN were a result of variations in their abilities to uptake specific nitrogen sources, and the residual amounts of these sources at the end of the growth phase varied between the WE and FWI strains on one hand and the NA, WA and SA on the other hand. The differentially transported N compounds included branched amino acids (leucine, valine and isoleucine) transported by the permeases Bap2p and Bap3p [71], the aromatic amino acids (tyrosine, phenylalanine) transported by the permeases Tat1p and Tat2p [72], glutamic acid transported by the permease Dip5p [79] and ammonium ions exclusively transported by the Mep1p, Mep2p and Mep3p permeases [27–28]. The variations the capacities of these strains to import these nitrogen sources may be due to mutations in the coding sequences that modulate the activities of the permeases or to differences in the expression pattern of genes encoding for these transporters. Comparison of the coding sequences and reciprocal hemizygosity analysis for the candidate genes exhibiting non-synonymous mutations allowed us to rule out the contribution of these mutations to the differences in the abilities of the strains to import nitrogen compounds.

The comparison of the gene expression profiles of strains exhibiting low or high capacities to uptake nitrogen sources during the growth phase revealed things including substantial differences in the levels of expression of a wide range of permeases. In particular, genes encoding for transporters of ammonium and branched amino acids were overexpressed in the WE and FWI strains. This indicates that an increased expression of the genes encoding for these permeases is likely responsible for the more efficient uptake of ammonium, phenylalanine, valine, acid glutamic, isoleucine, leucine and tyrosine by the WE and FWI yeasts. These variations are correlated with the fact that yeast strains are highly differentiated regarding their preferred nitrogen sources [43]. Overall, these variations in the consumption capacities for some nitrogen sources may be related to differences in the adaptation capacities between wine and non-wine strains to the wine environment. For example, the WE and FWI strains showed strong consumption of ammonium, which is the major nitrogen source present in the sugar cane and wine fermentation media. In addition, alanine is the only amino acid consumed to a large extent by the non-wine strains (NA, WA and SA). Alanine is the major nitrogen source in Chinese rice wine [13–14], and its concentration is dramatically different in wine must. Likewise, it appears that this medium is poor in ammonium ions, resulting in a lower consumption by the SA strain compared to WE [39]. However, these observations cannot be applied to the NA and WA strains because the nutritional compositions of their natural environments are difficult to quantify. Strains appear to consume large amounts of the nitrogen sources they find in their original environments, suggesting that nitrogen metabolism plays an important role in the adaptive evolution of strains of *S*. *cerevisiae* [80].

It seems that a greater ability to consume nitrogen is one of the factors involved in the adaptation of wine-type strains to their environment. This observation is correlated with the fact that a better capacity to uptake nitrogen compounds impacts the yeast fitness [12] and thus the ability to adapt to the environment. These differences in adaptation capacity between wine and non-wine strains to a wine fermentation medium are visible in the transcriptomic data, which reflect two distinct behavioral patterns.

The transcriptomic analysis between the strains from wine (WE) and non-wine (SA) environments revealed they had different strategies during the cellular growth and stationary phases. During the growth phase, the non-wine strain shows a strong cellular stress state in response to the environmental conditions [81]. Functional analysis by hemizygous construction conducted on the *RPI1* gene revealed that cellular stress has a negative impact on the nitrogen consumption capacity of yeast, which can explain the low nitrogen consumption capacity of the non-wine strains (NA, WA, and SA). In addition, this cellular stress state highlights the weak attributes of the non-wine strains and demonstrates the difficulties these strains face in adapting to the wine fermentation conditions [82]. This important period prior to growth initiation could explain the behavior of the strain observed at the end of cell growth. If the non-wine strain failed to store a sufficient amount of nitrogen by the end of the growth phase, the strain will express nitrogen permeases regulated by the NCR and SPS systems, and its energy will be directed toward cell proliferation. In contrast, the behavior observed for the wine strain (WE) during the growth cellular phase is characteristic of a strong cellular metabolism and indicates this strain is well-suited to wine fermentation conditions [43]. This adaptation performance is accompanied by significant nitrogen permease expression, which explains the greater nitrogen consumption of the wine-type strains (WE and FWI), and their ability to obtain a sufficient amount of cellular nitrogen to ensure optimal functioning of the organism during the fermentation process. This behavior is correlated to the behavior observed at the end of growth where the cells will adjust patterns of gene expression and protein activity to optimize metabolism. Yeasts direct their energy into biomass production by modifying the nitrogen cellular pool through the amino acid biosynthetic processes and the production of DNA/RNA building blocks with the purine nucleotide biosynthetic pathway, and an important portion of the observed genes are regulated by the GAAC pathway [83–84].

Notably, the transcriptomic comparison observed at the end of the growth phase revealed interesting information related to variations in nitrogen sensing signaling systems between the wine and non-wine strains. On one hand, the non-wine strain presented an overexpression of genes directed by the SPS and NCR systems, which are both regulated by the TOR complex 1 (TORC1) [33]. One the other hand, the wine strain revealed an overexpression of genes regulated by the GAAC pathway. These differences in genes expression effectively reveal two different regulatory behaviors with respect to nitrogen consumption.

Indeed, the differences in fermentation performances under wine conditions reflect the variations between the wine and non-wine strains on the medium adaptation, which could be explained by the distinct compositions of their original environments. The FWI and WE strains are typically usually used in the same food industry processes in which the YAN concentrations are very high [75]. These strains showed a similar fermentative behavior that suggests they are better adapted to this particular stress during the fermentation process. Inversely, the NA, WA and SA strains show the least adapted fermenting behavior to a standard winemaking medium. On one hand, the comparison of the kinetic profiles of the five strains confirmed that YAN consumption capacity is associated with variations in fermentation performances as previously demonstrated [85–86]. One the other hand, our results confirm that the capacity of yeasts to consume YAN has a substantial impact on biomass production, as previously reported [18].

The different strains used in this study have genetics variations [10] even if industrial strains are genetically grouped based on their industrial origin [11], which corresponds to how they impact human activity. The *S*. *cerevisiae* strain is not adapted to a particular environment, but yeasts have strong abilities to survive in a wide range of conditions [87]. Thus, the behavior observed in the non-wine strains depends more on the nitrogen composition of the medium, which is a challenge faced by the strain, rather than their genetic differences. Finally, the behavioral differences observed between the wine and non-wine strains can be a result of trait selection associated with the nitrogen substrates present in wine fermentation medium [6; 88–89].

## Conclusion

In this study, we investigated the metabolic and molecular basis for the differences in nitrogen consumption between strains. Using strains exhibiting extremely diverse nitrogen consumption profiles, we characterized this phenotype based on the variability between strains in their abilities to consume different quantities of YAN during the fermentation process. We determinate that variations in nitrogen consumption capacity between strains are associated with different adaptation capacities of the strains for the winemaking environment. Thus, the efficiency of nitrogen consumption seems to be involved in the ability of the strains to adapt to the winemaking environment. It would be interesting to know if variations in nitrogen consumption capacity reflect differences in the nitrogen metabolic requirements between the wine and non-wine strains. Further investigations are now in progress to identify the cause of this variability by exploring the management of the nitrogen anabolic requirements between these strains.

## Supporting information

S1 Table CO_2_ lost (g/L) during the fermentations processes using the NA, WA, SA, FWI and WE strains. (XLSX)

S2 Table HPLC results (mg/L) from samples collected during the fermentation processes. (XLSX)

S3 Table Nitrogen consumption (mg/L) of the reciprocal hemizygotes for *TAT2*, *DIP5* and *RPI1*. (XLSX)

S1 Fig Reciprocal hemizygosity analysis on the *TAT2* and *DIP5* genes. The YAN consumed is expressed as a percentage based on the amount of nitrogen consumed by the yeasts by the end of fermentation. Mean values and mean standard errors were calculated from three replicates. The SEM is indicated by vertical error bars. Values marked with an asterisk are significantly different (p<0.05).
(TIFF)

S2 Fig Comparisons of the physiological parameters of the five strains. (A) Comparison between the fermentation profiles of the five yeast strains with diverse YAN consumption profiles. The axes show the changes in the specific CO_2_ production rate with fermentation time on SM465. (B) Comparison between the dry masses of the strains at the end of fermentation. (C) Comparison of the protein contents of the yeast cells of the different strains at the end of fermentation. Mean values and standard errors of the mean (SEM) were calculated from three replicates. The SEM is indicated by vertical error bars. The values are expressed in g L^-1^. Values with different superscripts are significantly different (p<0.05).
(TIFF)
