# Supplementary figures and images for: Adaptability of the Saccharomyces cerevisiae yeasts to wine fermentation conditions relies on their strong ability to consume nitrogen
Source: PLoS One. 2018 Feb 12;13(2):e0192383. doi: 10.1371/journal.pone.0192383 (PMC5809068; doi:10.1371/journal.pone.0192383)

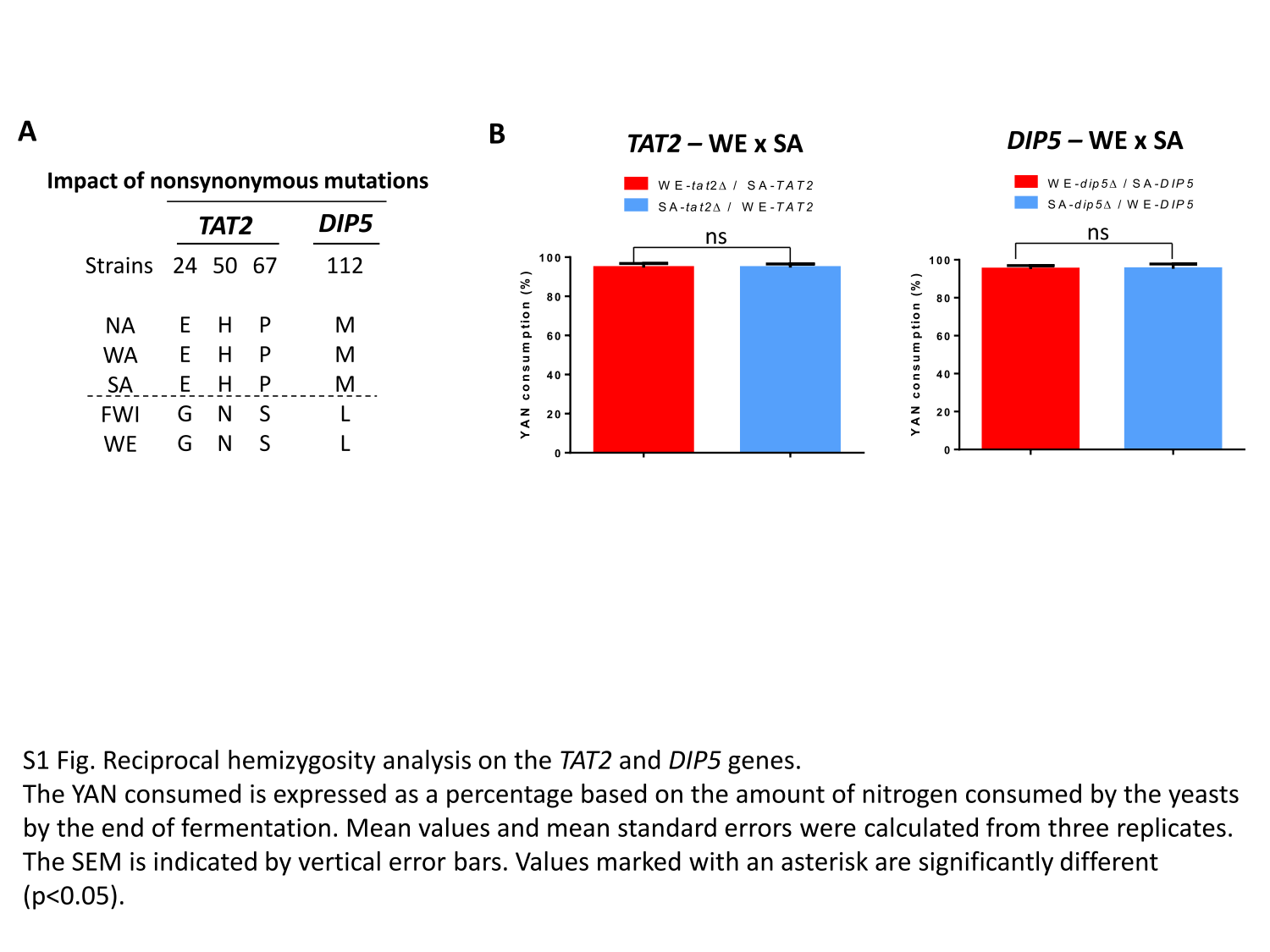

Supplement: S1 Fig — The YAN consumed is expressed as a percentage based on the amount of nitrogen consumed by the yeasts by the end of fermentation. Mean values and mean standard errors were calculated from three replicates. The SEM is indicated by vertical error bars. Values marked with an asterisk are significantly different (p<0.05). (TIFF) [file pone.0192383.s004.tiff]

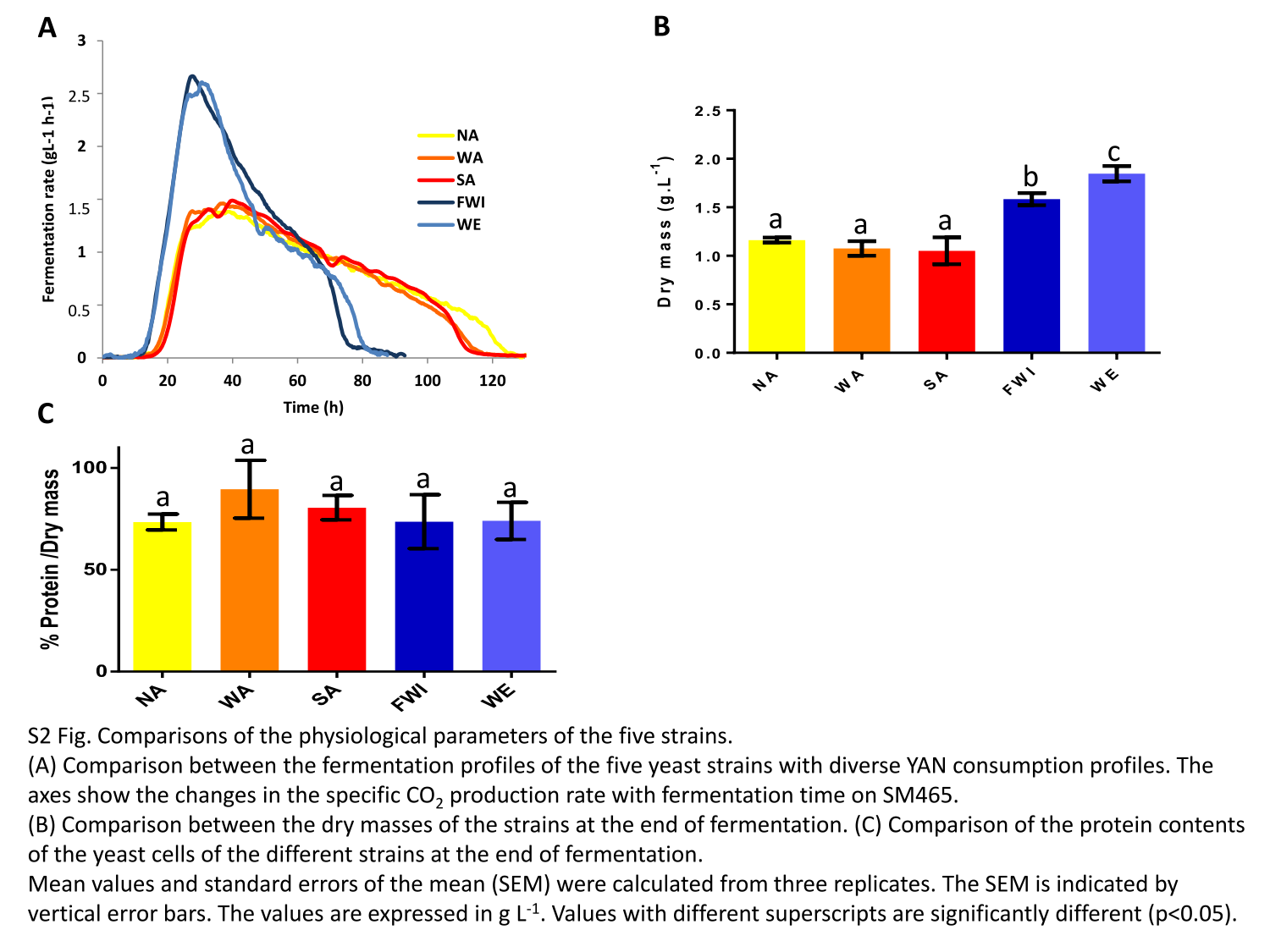

Supplement: S2 Fig — (A) Comparison between the fermentation profiles of the five yeast strains with diverse YAN consumption profiles. The axes show the changes in the specific CO2 production rate with fermentation time on SM465. (B) Comparison between the dry masses of the strains at the end of fermentation. (C) Comparison of the protein contents of the yeast cells of the different strains at the end of fermentation. Mean values and standard errors of the mean (SEM) were calculated from three replicates. The SEM is indicated by vertical error bars. The values are expressed in g L-1. Values with different superscripts are significantly different (p<0.05). (TIFF) [file pone.0192383.s005.tiff]
